# Biomechanical comparison of intuity vs. perceval aortic bioprosthesis: apples & oranges or swings & roundabouts?

**DOI:** 10.1186/s13019-025-03567-8

**Published:** 2025-09-17

**Authors:** Konstantina Spetsotaki, Matthias Menne, Ajay Moza, Shahram Lotfi, Ali Aljaloud

**Affiliations:** 1 Present Address: Department of Thoracic Surgery, University Hospital Zurich, Raemistrasse 100, Zurich, 8091 Switzerland; 2 Department of Cardiovascular Surgery, RWTH University Hospital Aachen, Aachen, Germany; 3 Department of Cardiovascular Engineering, Institute of Applied Medical Engineering, Helmholtz Institute, Medical Faculty, RWTH Aachen University, Aachen, Germany; 4 Rhein-Maas Hospital, Department of Cardiology, Nephrology and Internal Intensive Care, Würselen, Germany

**Keywords:** Rapid deployment, Sutureless aortic valve replacement, Intuity, Perceval

## Abstract

**Introduction:**

Rapid deployment (RD) and sutureless (SU) aortic valve replacement (AVR) are established strategies with proven benefits and ongoing evolution. In this study, we compare the clinical results and technical attributes of RDAVR and SUAVR with the two most commonly applied bioprostheses, the Edwards Intuity Valve System and the Perceval sutureless.

**Methods:**

*N=*19 patients with Intuity AVR were matched to *N=*19 with Perceval AVR from 2014 to 2020 at University Hospital Aachen, RWTH. Preoperative and postoperative data were compared. OSIRIX DICOM software was used for 4D stent analysis 30 days post−surgery. Additional in vitro evaluation of the Perceval and Intuity Valve Systems’ radial forces was conducted.

**Results:**

In−hospital and 30−day mortality was 2/19(10.5%) in both groups. Patients in the Perceval group had higher MPG score value than those in the Intuity group (13.96 vs. 10.70; *p*=0.041). Patients in the Perceval group had significantly higher mean values of postoperative PPG than those in the Intuity group (26.34 vs. 19.52, *p*-value = 0.018). The Intuity group showed higher roundness in CT analyses after AVR and higher radial forces than the Intuity group during the in−vitro testing.

**Conclusion:**

We report interesting differences regarding the biomechanical behavior of the stents’ ovality and radial forces of the two prostheses. However, the early postoperative clinical outcome remains comparable. Further studies with larger cohorts and long−term mechanical analysis are needed for deeper insights into this complex entity.

## Introduction

Sutureless (SU) and rapid deployment (RD) aortic valve replacement (AVR) have emerged as innovative modalities that play a pivotal role in the field of surgical aortic valve replacement for the management of structural heart valve disease [1]. The sutureless aortic valve replacement (SUAVR) with Perceval and the rapid deployment aortic valve replacement RDAVR with Edwards Intuity™Elite are proven to be efficient and safe [1], promising the advantages of thetranscatheter aortic valve replacement (TAVR) procedures, also providing a plethora of benefits of a conventional surgical approach, such as the removal of the native valve along with the calcifications, with advantage beyond the conventional technologies, even in elderly, frail, high-risk, redo, bicuspid aortic valve, with application even in some porcelain aorta cases [2, 3]. Several studies indicate that the hemodynamic performance of the Intuity valve surpasses that of conventional AVR. However, there are only a few studies comparing the Intuity and Perceval valve systems [4], and the majority of those only focus on clinical outcomes with no studies comparing biomechanics, geometric imaging, and postoperative adaptation. The durability and performance of a bioprosthesis depend on a myriad of factors, and structural dynamics, including the intricate interaction between the bioprosthesis components, the stent, and the native aorta [5]. We analyzed and compared the Intuity vs. Perceval Valve Systems, through in vitro testing, in vivo 3D cardiac CT stent analysis, and clinical outcomes after SURD-AVR.

## Materials and methods

### Patient cohort and clinical outcome analysis

This is a single−center observational study with radiological, biomechanical, and clinical comparative aspects of the Intuity Elite/Edwards Lifesciences Inc., Irvine, CA, USA) and the Perceval (Corcym S.r.l., Italy) bioprosthesis. Patients who received AVR with Intuity and Perceval bioprostheses from 2014 to 2020, in the Cardiothoracic Surgery Department of the University Hospital Aachen, RWTH were analyzed. All patients were operated by the same team of expert surgeons following the same protocols. The total sample size was *N=*38 with 19 patients in each group. Patients who received the Intuity RDAVR were matched with similar risk profile patients from the Perceval SUAVR. The matching process used variables like age, height, BMI, STS−score, EuroscoreII, preoperative thrombocytes, and lactate dehydrogenase (LDH). Preoperative and postoperative clinical data were compared. Variables are presented in Table 1. The study was approved by the local ethics board (Ethics Commission RWTH Aachen, IRBP 69 10/2014, and EK 151/09−Version−1.3) and waived informed consent due to the study’s retrospective nature.

**Table 1:** Baseline, operative, and postoperative data

Variable	Intuity (*N=*19)	Perceval (*N*=19)	*p* value
Age (years)	79.53 ± 7.17	77.53 ± 3.59	0.142
Height (m)	1.69 ± 0.09	1.67 ± 0.09	0.632
Weight (kg)	79.74 ± 11.55	79.50 ± 14.36	0.957
BMI	28.02 ± 4.49	28.11 ± 3.31	0.943
Thrombocytes pre-op (× 10^9^/L)	261.37 ± 66.22	261.53 ± 54.19	0.994
LDH pre−op (U/L)	254.84 ± 129.27	234.06 ± 79.07	0.562
Euroscore II	2.19 ± 0.78	2.18 ± 0.69	0.934
STS score	1.66 ± 0.73	1.59 ± 0.93	0.826
Female, n (%)	11 (58)	10 (52.6)	0.746
COPD, n (%)	2 (10.5)	3 (15.8)	0.631
Diabetes, n (%)	5 (26.3)	6 (31.6)	0.721
NYHA > II, n (%)	6 (31.6)	2 (10.5)	0.111
PAD, n (%)	1 (5.3)	1 (5.3)	1.000
HLP, n (%)	4 (21.1)	4 (21.1)	1.000
Bicuspid aortic valve	-	-	
Severe Aortic Stenosis	19 (100.0)	19 (100.0)	0.200
Mild Aortic Regurgitation	5 (26.3)	7 (43.8)	0.200
CPB in Minutes	147.00 (130.00–174.00)	105.00 (97.50–146.00)	0.033
Cross−clamp time (minutes)	107 ± 3	90 ± 10	0.031
Thrombocytes post-op (× 10^9^/L)	178.63 ± 45.81	171.50 ± 54.39	0.686
LDH post−op (U/L)	373.00 ± 300.00	412.59 ± 81.63	0.332
Echocardiographic Parameters			
MPG (mmHg)	10.70 ± 3.45	13.96 ± 4.59	0.041
PPG (mmHg)	19.52 ± 5.88	26.34 ± 8.38	0.018
Velocity Ratio	0.48 ± 0.09	0.36 ± 0.11	0.008
VTI	0.80 ± 0.17	0.59 ± 0.17	0.005
ET (ms)	250.00 (241.00–262.00)	256.00 (218.00–279.25)	0.723
AT (ms)	75.00 (72.00 ± 80.00)	62.00 (41.00–73.00)	0.025
TAPSE (mm)	15.85 ± 2.21	15.32 ± 4.80	0.356

IVSD (cm) 0.8 (0.7–0.9) 15.00 (12.00–16.00) < 0.001

*BMI* Body mass index, *EuroSCORE II* European System for Cardiac Operative Risk Evaluation, *STS score* Society of Thoracic Surgeons score, *COPD* Chronic obstructive pulmonary disease, *NYHA* New Hork Heart Association, *PAD* Peripheral arterial disease, *HLP* Hyperlipoproteinemia, *LDH* Lactate dehydrogenase, *MPG* Mean pressure gradient, *PPG* Peak pressure gradient, *VTI* Velocity time integral, *ET* Ejection time, *AT* Acceleration time, *TAPSE* Tricuspid annular plane systolic excursion, *IVSD* Interventricular septum thickness

### Statistical analysis

For all numerical variables, a test of normality was performed using the Shapiro–Wilk test. All numerical variables that are normally distributed are presented as mean ± standard deviation. Values that are not normally distributed are presented as median (IQR). Patients who received the Intuity RDAVR during the study period were matched with similar risk profile patients from the Perceval SUAVR. The matching process used baseline variables such as age, height, BMI, STS−score, EuroscoreII, preoperative thrombocytes, and LDH. In order to compare the difference between the two groups, independent t – test for normally distributed non−repeated continuous variables and Mann–Whitney U−test for non−normally distributed continuous variables were performed. Comparison in each group for preoperative (pre) and postoperative (post−op) measurements was performed using a Paired t−test. Categorical variables are presented as frequencies and percentages (n,%). Chi−square test (X2) was used to compare the categorical variables associated with each group (Intuity, Perceval). *P*-values <0.05 were considered statistically significant. All analyses were performed using the statistical software SPSS 29.0 (IBM Statistics).

### Aortic prosthesis

The Perceval Valve is a bovine−derived pericardial prosthesis mounted on a self−expanding Nitinol stent. In the absence of anchoring circumferential annular sutures, these rapid deployment sutureless prosthetic devices are secured in place by the radial force (RF) exerted by the stent component on the patient’s aortic annular and root plane [4]. The main characteristic of Nitinol frames is their shape memory, which causes them to seek their expanded shape as a function of temperature. They exhibit a certain degree of hysteresis in their RF profile. The RF during crimping is called radial resistive force and is generally higher than the RF values during expansion, which is called chronic outward force. This characteristic behavior of Nitinol stents is known as Stress Hysteresis or"biased stiffness" [6–8]. Edwards Intuity™ Elite is a bovine aortic prosthesis crafted to emulate the widely−utilized Perimount Magna Ease aortic prosthesis, featuring a stainless steel stent and a fabric sheath. Stainless steel (SS) is an iron−based metal alloy with at least 10.5% chromium (Cr), which shows good corrosion resistance, high endurance strength, excellent mechanical stiffness, adequate biocompatibility, relatively low manufacturing cost, and easy fabrication, which makes it one of the most widely used materials in medical engineering [9, 10]. SS alloys are used for permanent implants (bone implants, stents, dental implants, etc.) and temporary implants as well. In the United States, it is estimated that almost 60% of surgical implants are made of SS [11]. Metallic biocompatible materials, particularly stainless steel and titanium composites, are among the most widely used biomaterials for bioengineering applications due to their excellent mechanical behavior and biocompatibility [6, 12].

### In vitro radial force analysis

In vitro stent evaluation of the Perceval and Intuity valve systems were performed with a commercial radial force tester, commonly used for regulatory testing (RX650, Machine Solutions, Flagstaff, Arizona, USA). It consists of a crimping mechanism with 12 triangular jaws that open and close around the stent, comparable to a camera lens, and allow for its expansion. The RX650 measures RF of self−expanding and balloon-expandable heart valves and stents, conforming to ISO 5840–3, ISO (2003). ISO 25539–1 and ISO (2000), ISO/TS 15539 (Cardiovascular, Endovascular Implants and Prostheses, International Organization for Standardization, Geneva, Switzerland), and is FDA approved (2010 Non-Clinical Tests and Recommended Labeling for Intravascular Stents and Associated Delivery Systems, MD, USA). Officially, the tester’s measurement accuracy is noted as 0.06%. The RF measures the stent’s resistance to radial deformation, replicating in vivo conditions accurately, giving insights into valve performance and behavior in vivo, and is common in heart valve design [8]. We inserted the same size of Intuity and Perceval of 27 mm, to compare their RF profiles. The machine was programmed to gradually crimp, press down, and compress from 35 to 25 mm, while consistently measuring the exerted radial force. Moreover, to eliminate the possible bias of the Perceval test process, considering the temperature−sensitive characteristics of the nitinol stent of the Perceval valve system, whose radial force is greatly influenced by temperature conditions, we carried out the testing in a climate chamber at 37 ± 1 °C to replicate the in situ conditions. Each Perceval valve underwent three rounds of testing, with them resultant average being presented herein. The Intuity valves were only tested once since permanent deformation may occur and recurring testing would therefore impact the accuracy of the measurement.

### CT imaging analysis

All cardiac CT follow−up images were performed 30 days post−AVR in our institution using the same standardized protocol. We applied the OSIRIX DICOM software for 3D and 4D analysis of the stents’ ovality and geometry in these CT reports. The software is an advanced, efficient, and reproducible tool already used for preoperative evaluation of aortic diseases, spinal cord vasculature imaging, planning aortic procedures, and follow-up after stent interventions [13, 14].

**Fig. 1:**
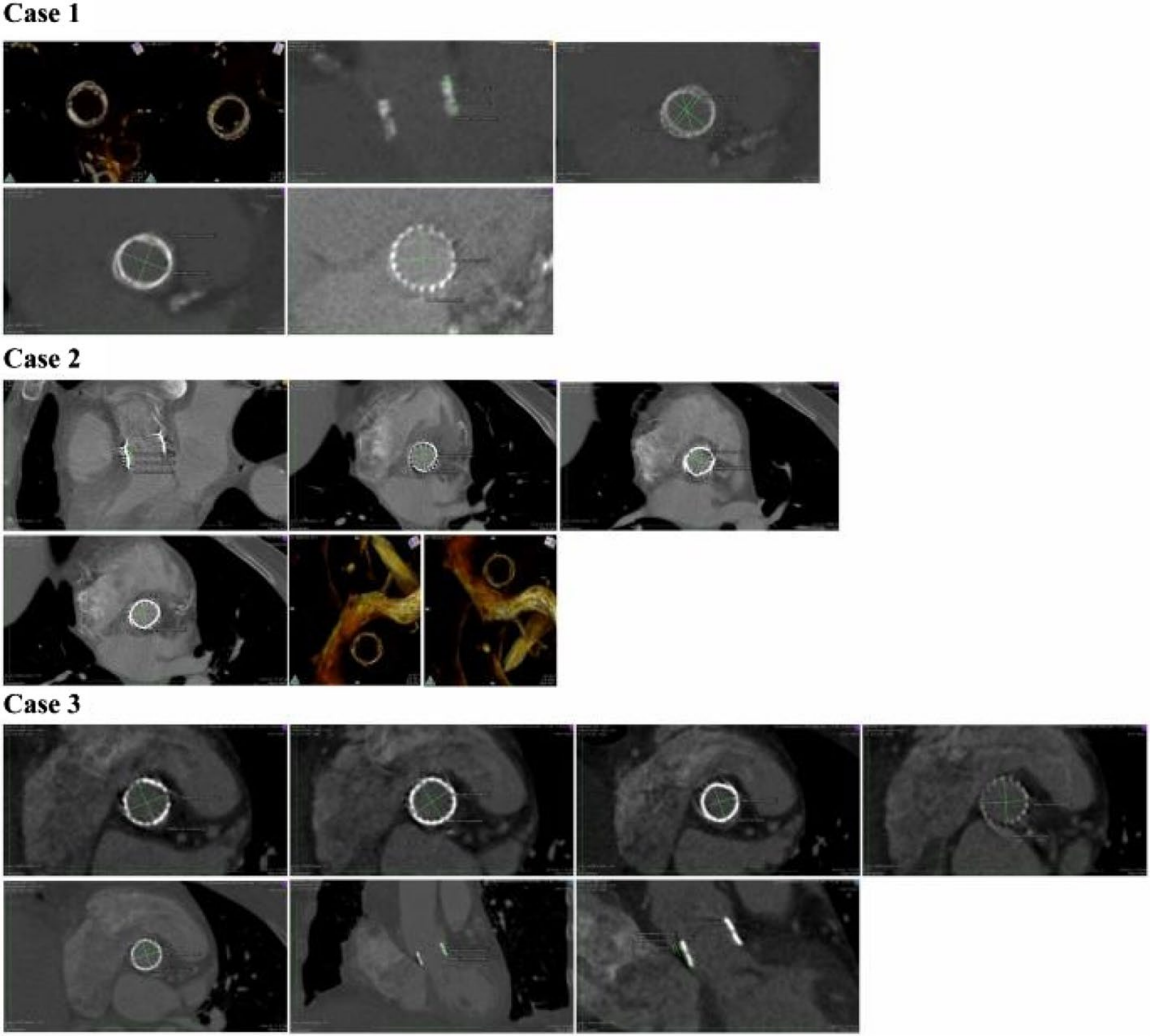
CT-Follow-up of Cases that received RDAVR with the Intuity image series obtained with OsiriX ™ providing us a pseudo-realistic three-dimensional depiction of the human body, highlighting the aortic bioprosthesis in situ with different views at different levels of the valve level, with the actual measurements taken on an appropriately reformatted CT scan

**Fig. 2:**
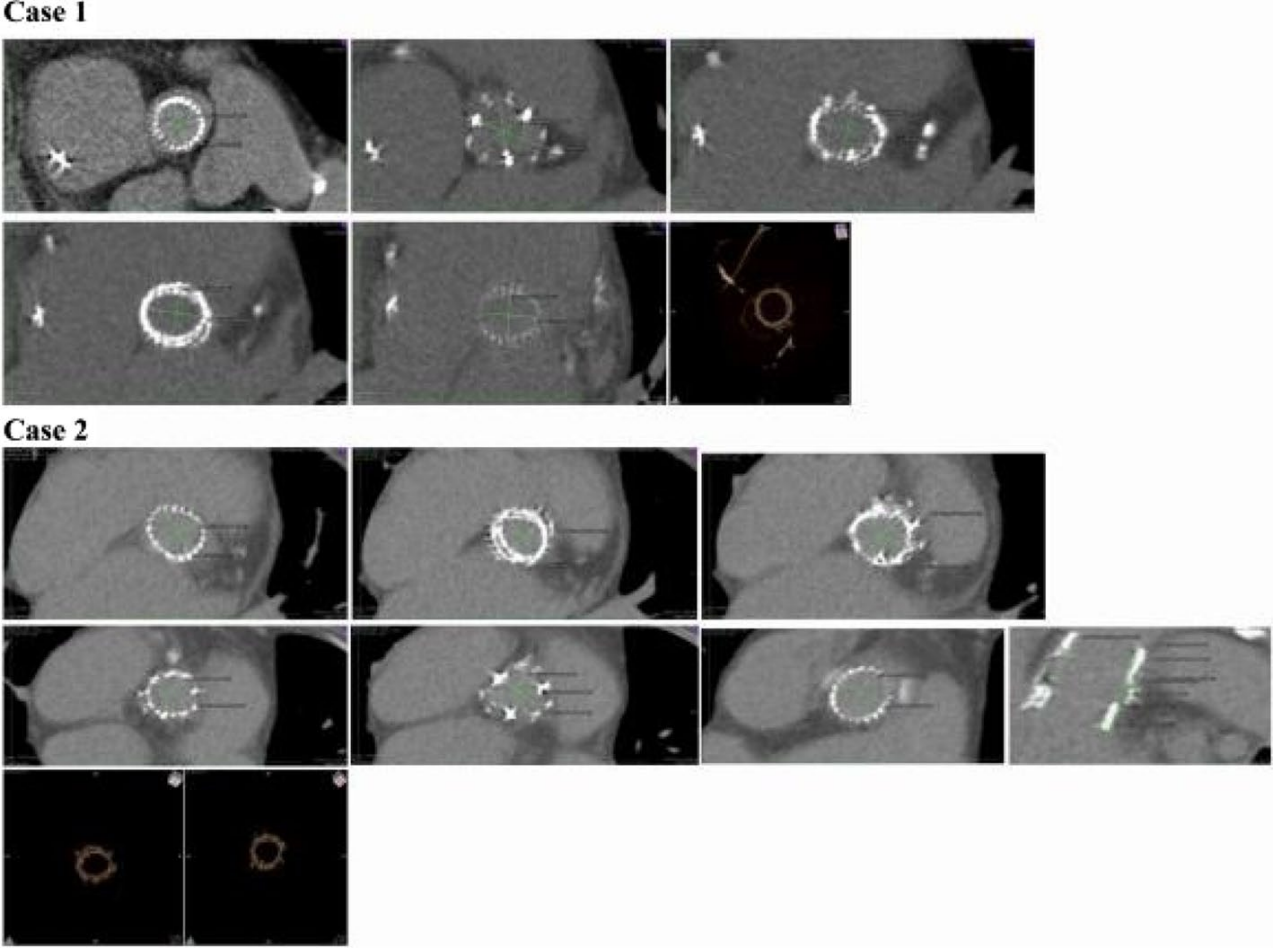
CT follow-up of Cases that received SUAVR with the Perceval image series obtained with the OsiriX™ providing us a pseudo-realistic three-dimensional depiction of the human body highlighting the aortic bioprosthesis in situ with different views at different levels of the valve, with the actual measurements taken on an appropriately reformatted CT scan

The proposed OsiriX software was applied for the radius and ovality evaluation of the bioprosthesis by an expert unaware of the clinical outcomes. Ovality assessments were conducted at the level of the annulus and the distal extremity of the skirt. The best CT 2D multi−planar reconstruction images were stored and evaluated by another expert for stent radius and ovality (Figs. 1 and 2).

Stent ovality O, served as a measure of stent deformation. The ovality percentage can be computed using the lengths of both the major and the orthogonal minor axis of an ellipse using the following formula:

$$0=\frac{2*({L}_{max}-{L}_{\perp })}{{({L}_{max}+L}_{\perp })}* 100{\%}$$

The ovality of a circle is 0 and the higher the ovality value, the more oval the shape. All patients received a postoperative transthoracic echocardiogram at the time of hospital discharge. All echocardiograms were conducted and analyzed by expert echocardiographers according to the European and American Society of Echocardiography Guidelines [15, 16]. The research was approved by the local institutional review board IRB (EK 151/09 version 1.2.3) and complies with the principles outlined in the Declaration of Helsinki.

## Results

### Baseline characteristics

The two groups differed in New Hork heart Association (NYHA) grade, with *N*=6 160 (31.6%) in the Intuity group showing NYHA > II, vs. *N*=2 (10.5%) in the Perceval group presented with NYHA > II X^2^ (2, *N*=35):8.887, *p*-value=0.012. COPD was present in 10.5% of the Intuity and 15.8% of the Perceval group, respectively. Diabetes was present in 26.3% of the Intuity and 31.6% of the Perceval group, respectively. The two groups did not differ significantly regarding preoperative diagnosis such as COPD and Diabetes. No patients showed severe aortic regurgitation (AR) prior to surgery. There was no significant difference in the incidence of AR between the two groups. Detailed data are presented in Table 1.

### Postoperative results

Postoperative outcomes and the compilation of complications observed are delineated in Table 2. Operative mortality was 0%. One case within the Intuity group needed re−exploration in the early postoperative period following a cardiac arrest and ROSC, in the absence of any indications related to the prosthesis. All−cause mortality in both groups was 10.5%. *N=*6/19 (31.6%) of the Intuity and *N=*7/19 (36.8%) of the Perceval had an uneventful postoperative course. *N=*3/19 (17.5%) in Intuity and 1/19 (5.3%) in the Perceval group needed a permanent pacemaker impantation (PPI). Additionally, in the Intuity group, there was a significant reduction of thrombocytes postoperatively [17]. The mean LDH difference between pre-op and post−op values was −118.15 (± 100.49, CI (−166.59, −69.72), *p*-value <0.001, indicating a significant LDH elevation post−RDAVR. While, in the Perceval group, there was a significant decrease in the Thrombocytes after surgery: the mean difference between pre-op and post-op Thrombocytes values was 87.07 (± 85.81, CI (37.52, 136.62), *p*-value < 0.001. The mean difference between pre-op and post-op LDH was −175.35 (± 84.60, CI (−218.85, −131.85), *p*-value < 0.001, meaning that LDH was significantly increased post-SUAVR.

**Table 2:** Postoperative complications

Postoperative complications Intuity (*N=*19) Perceval (*N=*19) *p*-value			
None	6 (31,6)	7 (36.8)	> 0.005
Myocardial Infarction	0 (0.0)	0 (0.0)	> 0.005
Stroke	0 (0.0)	0 (0.0)	> 0.005
AF alone	1 (5.3)	2 (10.5)	> 0.005
AV-Block III with PPI, POD, AKI	3 (17.5)	0 (0.0)	> 0.005
Pneumonia, POD, Other arrhytmias	2 (10.5)	2 (10.5)	> 0.005
AV Block III with PPI, RV-failure	0 (0.0)	1 (5.3)	> 0.005
Pneumonia	1 (5.3)	1 (5.3)	> 0.005
POD alone	1 (5.3)	1 (5.3)	> 0.005
Other arrhythmia, RV−Failure, VTs	1 (5.3)	0 (0.0)	> 0.005
AF, Delir, RV−Failure, AKI, Sepsis	1 (5.3)	0 (0.0)	> 0.005
POD, AKI	1 (5.3)	1 (5.3)	> 0.005
AF, Pneumonia	0 (0.0)	1 (5.3)	> 0.005
POD, wound complications	0 (0.0)	1 (5.3)	> 0.005
AF, POD, Pleural effusions, other arrhythmias	0 (0.0)	1 (5.3)	> 0.005
AF, other arrhythmias	0 (0.0)	1 (5.3)	> 0.005
CPR with ROSC, Re−Exploration	1 (5.3)	0 (0.0)	> 0.005
AF, pneumonia	1 (5.3)	0(0.0)	> 0.005
In−hospital mortality	1 (5.3)	1 (5.3)	> 0.005
Cardiac Cause 30−day mortality	1 (5.3)	1 (5.3)	> 0.005
All-cause 30-day mortality	2 (10.5)	2 (10.5)	> 0.005
Moderate PVL	1 (5.3)	2 (10.5)	> 0.005
Severe PVL	0 (0.0)	0 (0.0)	> 0.005

*AF* Atrial fibrillation, *VT* Ventricular tachycardia, *POD* Postoperative delirium, *RV* Right ventricular, *AKI* Acute kidney injury, *CPR* with *ROSC* Cardiopulmonary resusscitation with return of spontaneous circulation, *PVL* Paravalvular leakage, *RV* Right ventricle

Based on the postoperative echocardiographic measurements, as described in Table 1, there was a significant difference between the two groups (*p*-values < 0.05). Perceval group had a higher mean MPG score value than the ones in Intuity (13.96 vs. 10.70 respectively). Patients in the Perceval had significantly higher mean PPG than in Intuity group (26.34 vs. 19.52, *p*-value=0.018). There was a significant difference between the two groups’ velocity ratio mean values, with patients in Intuity having higher velocity ratio than the ones in Perceval (0.48 vs. 0.36 respectively, *p*-value=0.008). Intuity group showed significantly higher VTI mean values than the Perceval group (0.80 vs. 0.59 respectively, *p*-value=0.005), and significantly higher mean AT values than the ones in Perceval (Medians were 75.00 vs. 62.00 respectively, *p*-value=0.025).

### CT analysis results

The stent ovality for the Intuity valve at the annulus level ranged from min 0.00%, to max 0, 01%. Stent ovality at the distal edge of the stent was in a range of 0.7% to 19% for the Intuity valve. For the Perceval valve, a range of ovality from 14 to 45% was recorded. Some examples are presented in Figs. 1 and 2.

### Radial force results

**Fig. 3:**
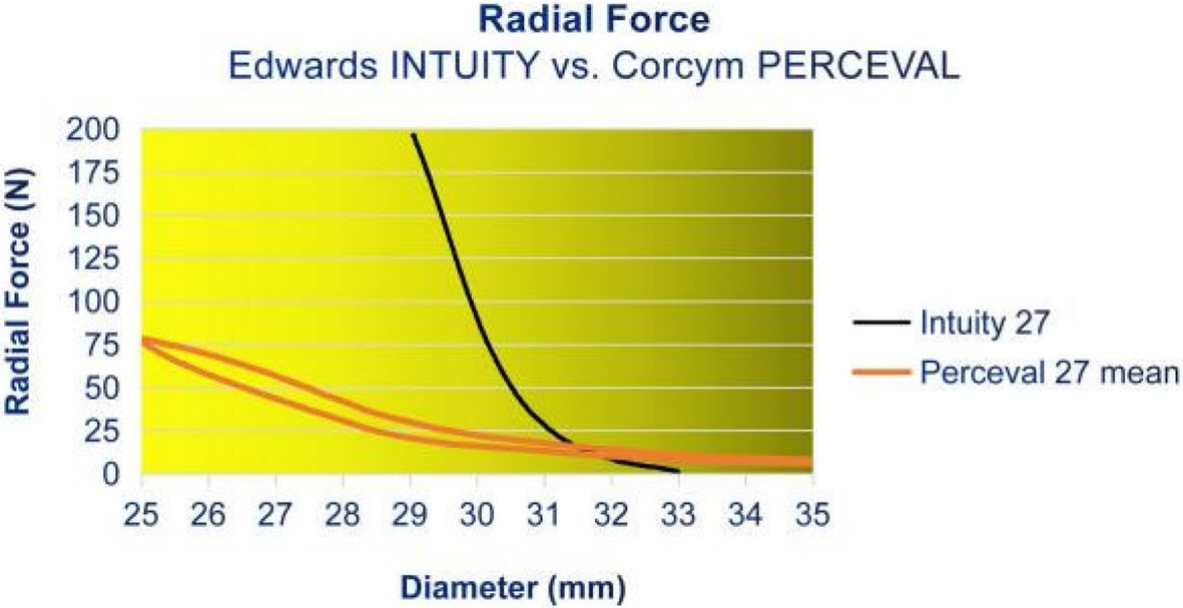
Radial Force Profiles: Intuity vs. Perceval

For the Intuity prosthesis, the RF increased gradually during the RF testing process, starting at a diameter of 33 mm during compression. Below a compression of a diameter of 31 mm, the RF increased quickly until it reached 200 N at 29 mm. The test had to be halted upon reaching 200 N, as this exceeded the load cell’s maximum capacity in the radial force tester and risked damaging the equipment. The RF of the Perceval valve increased gradually until a diameter of 29 mm and increased more steeply until it reached its maximum RF of 78.1 N at 25 mm of diameter. These results indicate that the Intuity valve exhibits a higher degree of rigidity and is less susceptible to deformation compared to the Perceval bioprosthesis. Figure 3 shows the average results of our measurements.

## Discussion

Intuity and Perceval are the two widely applied SU−, RD−AVR bioprostheses worldwide, having gone through a long journey of improvements, milestones, and evolution. A plethora of clinical studies compare the two valve systems, Intuity and Perceval, proving comparable clinical results by terms of early postoperative outcomes [4]. However, to the best of our knowledge, our study, although presenting a small cohort, is the first of its kind to compare not only the early clinical outcomes, but also their biomechanical adaptation, ovality, and stent performance after surgery, and their in−vitro RF profile testing. Beretta et al. showed in a multicenter registry reporting 4,695 patients, and comparing Intuity vs. Perceval prosthesis in isolated and or combined AVR procedures, the Intuity showed superior hemodynamic outcomes with significantly lower transvalvular gradients after surgery, compared to the Perceval alternative, in both isolated and combined AVR cases [18]. This is consistent with our echocardiographic clinical results. Additionally, the Intuity alternative was related to lower rates of aortic regurgitation [18]. The Perceval valve was associated with reduced operative times. The Intuity valve distinguishes itself from other RD and conventional valves by yielding superior outcomes, such as a significant reduction in mortality, an increase in the durability of the valve, and a marked decrease in both mean and peak transvalvular pressure gradients [19]. Additionally, Intuity AVR was carried out effectively in a diverse patient population, addressing various conditions, including bicuspid aortic valve, isolated aortic regurgitation, and infective endocarditis, demonstrating exceptional clinical and hemodynamic improvements [20]. In a comparable report, total valve-related complications were 12% in the Intuity and 20.5% in the Perceval group, stroke rates of 12% in Intuity and 20% in the Perceval, and average peak and mean pressure gradients of 18 ± 9 mmHg and 10 ± 5 mmHg for the Intuity [21]. Despite a non-significant statistical tendency for higher peak pressure gradients in the Perceval group (22 ± 6 mmHg, *p*=0.057), the mean pressure gradients were similar to the Intuity (11 ± 4 mmHg, *p*=0.991). Moreover, a 9% PPI after Perceval and 8% need for PPI after Intuity SUAVR were reported [21]. These clinical findings are consistent with our clinical observations, although in our study the difference in MPG and PPG was significant. Echocardiographic studies comparing the initial and longer-term (up to 5 years) follow-up of the Perceval vs. the Intuity RDV (up to 12 months of follow-up) have consistently shown excellent echocardiographic performance in terms of pressure gradients and effective orifice area (EOA) [21]. Regarding the biomechanical characteristics and design, the primary distinguishing difference between the two prostheses lies in the fact that Intuity features a rapid deployment valve with a SS stent, utilizing three anchoring sutures through the annular nadirs, whereas Perceval offers a sutureless alternative devoid of any suture application, and consists of a nitinol stent. The Intuity valve features a valve annulus stent enveloped in a polyester sealing fabric [22]. Meanwhile, the Perceval valve system features a larger diameter compared to the specified annuli range, crafted to apply a radial force crucial for both valve sealing and anchoring post-implantation [22]. Comparative studies between the Edwards Intuity and Perceval sutureless valves primarily focus on their performance and postoperative results, with each valve having distinct characteristics and outcomes. Studies suggest that the Intuity valve offers favorable outcomes, with reduced procedural time and good early postoperative results, rapid recovery, and shorter hospital stays. On the other hand, the Perceval valve, particularly the Perceval PLUS, has been evaluated in several studies with larger patient populations [23]. To the best of our knowledge, this is one of the few studies to compare the two valve systems, and one of its kind to analyze an evaluation of not only the clinical but also the biomechanical aspects such as radial forces and ovality after AVR. There is a plethora of factors influencing the early and long-term valve performance after AVR. These encompass the design and mechanical characteristics of the prosthesis, the surgical technique, and the patients’ anatomy. It is proven that the ellipticity of the aortic annulus can influence the results of some prostheses [24]. The human aortic annulus is not perfectly circular; it rather exhibits an elliptical shape. Additionally, patients with aortic valve stenosis often have stiffer tissues than the average patient [25]. Sutured, stented valves enforce the human annular plane into its desired circular shape, providing proper long-term performance of the leaflets. Different stress patterns can cause different stress peak points on the leaflets, commissures, or stent of a bioprosthesis, triggering different valve degeneration patterns [26, 27]. The biomechanics of TAVI prostheses have been deeply analyzed through a plethora of studies [5, 27]. However, there are only a few studies regarding the prosthesis-annular plane adaptation in cases of SU-, RD-AVR. In our previous studies, we reported some observations of the Perceval bioprosthesis’s biomechanics using different techniques [17, 28].

Additionally, Auricchio et al. measured the eccentricity and stent configuration of bioprostheses, and proved that the eccentricity of the deployed stent drastically affects the valve closure and the coaptation of the leaflets, consequently the performance and durability of the valves [29]. A plethora of studies referred to bioengineering analysis of the aortic valve and prosthesis and have proved that the geometrical asymmetry-ellipticity of a stent is a crucial determinant of the central gap during diastole, caused by the malcoaptation of the leaflets and a flow regurgitation [5, 30]. Our 3D CT analysis revealed that the Intuity tends to maintain its circular shape, more than the Perceval prosthesis, which is susceptible to deformation, possibly due to its pliable nitinol structure. This represents the first application of this software for the 3D examination of the stents’ ovality and geometry during the postoperative monitoring following AVR, and there are no other comparable findings to juxtapose. We observed ovality patterns for the Intuity valve of almost 0%, equivalent to an almost 100% roundness that approaches that of a perfect circle. On the contrary, the Perceval stent tended to higher grades of ovality and deformation and remained less round in follow-up. Additionally, to the best of our knowledge, this is the first report of the SUAVR and RDAVR valve system’s RF profiles. Our in-vitro RF observations indicate that the Intuity valve demonstrates a superior level of stiffness, implying a decreased vulnerability to deformation in comparison to the Perceval prosthesis. The RF of the Intuity valve at 29 mm, which is 2 mm above its intended implantation range, is 2.5 times higher in force than the Perceval valve at the lower end. The Intuity valve may be even more rigid within its range, but the RF tester maxed out at 200 N. 200 N is equivalent to a 20 kg load on the stent frame, more than sufficient for its purpose. The RF of the Intuity bioprosthesis seems high enough for a circular contour in vivo, possibly explaining good results and low patient-prosthesis mismatch rates, even in patients with small aortic roots [31]. A study involving particle image velocimetry and hydrodynamic characterization, analyzing the in vitro flow model of Intuity Elite based on micro-computed tomography scans of the implanted Intuity and adjacent cardiac structures, showed its intra-annular inflow frame of the rapid deployment to create a wider left ventricular outflow tract. In vitro flow models were developed, showing that the Intuity’s design consequently decreases the pressure gradient across the valve and enhances overall hemodynamic function [32]. The RF of the Perceval prosthesis became quantifiable once the diameter reached 35 mm. It exhibited a steady rise beyond 25 N when the diameter was 29 mm. Below 29 mm, the escalation became more pronounced until the RF peaked at 78 N when the prosthesis was at its smallest intended diameter of 25 mm. That indicates that the Intuity valve exhibits a higher degree of rigidity, suggesting a reduced susceptibility to deformation compared to the Perceval prosthesis. Our in vitro RF test results are in concordance with our clinical real-life CT imaging observations and could also elucidate the findings of previous studies conducted by our team, which demonstrated stent deformation and the “flutter-by-effect” of the Perceval valve [28]. On the other hand, the RF profiles described above could be explained as a “double-edged sword” due to the fact that the lower rigidity of the nitinol Perceval stent offers other benefits, such as its easy application in Valve-in-Valve-TAVR procedures, easier and safer, primarily due to the distinctive features of the nitinol stent, radiopaque frame, and sinusoidal struts [33]. While the rigid Intuity SS stent, offering a sturdy and well-defined structure, durability, and harmonious hemodynamics, could explain the high incidence of high-grade AV-block with PPI need due to the higher mechanical stress on the surrounding conductive structures [33]. However, as already mentioned, multiple other factors like material, prosthesis size, deployment technique, decalcification sufficiency, skirt-stent distance, stent’s inflow expansion, valve positioning, aorta geometry, and patient’s anatomy could impact stents’ long-term adaptation and durability and should be taken into consideration [5]. We observed that the Intuity stent seems to be more rigid and to maintain its ovality, but applies stronger radial forces to the native tissues, while the Perceval stent appears to produce a lower RF pattern and be more susceptible to deformation; however, this mostly remains sub-clinical and did not manifest any early postoperative clinical deterioration in these patients at the time of the study, with the early postoperative clinical outcome remaining comparable between the two bio-prostheses. However, it has to be underlined that structural valve deterioration (SVD) develops progressively through classified stages 0–3, with the initial stages 0, and 1, presenting no hemodynamic alterations and being characterized by sub-clinical progressive lesions during imaging follow-up [5]. Our study consists of three parts: 1. the clinical observational retrospective part that analyzes the postoperative clinical outcomes after AVR, 2. the ovality evaluation via CT imaging follow-up analysis of the postoperative valve ovality in situ, and 3. the separate in-vitro testing of the RF profiles of the two valve systems. Although the clinical part presents certain limitations such as the retrospective nature of the analysis of the clinical results and the small sample size of patients, the strength, uniqueness and the primary focus of this study is based on the biomechanical comparison of the two valve systems and based on evidence of the RF testing and those exaclty are the strengths of this study.

## Conclusions

We report interesting new insights into the biomechanics of the Intuity and Perceval bio−prostheses. Each bio−prosthesis possesses unique merits, and both SUAVR and RDAVR technologies must continue to advance in order to compete with the improving TAVR technologies. Further studies with larger cohorts and long−term bio−mechanical and imaging observations are needed to gain a deeper understanding of the impact of different stents’ bio−mechanical dynamics, shape, and adaptation under various physiological conditions, and determine whether these significantly trigger the onset/progression of SVD and thrombosis on long−term, which remain the Achilles’ heel of all bio−prosthetic valve systems.

## Data Availability

No datasets were generated or analysed during the current study.

## Abbreviations

AR: Aortic regurgitation AV atrioventricular
AVR: Aortic valve replacement
COPD: Chronic obstructive pulmonary disease
CPB: Cardiopulmonary bypass Cr chromium
EOA: Effective orifice area
HVD: Heart valve disease
HLP: Hyperlipoproteinaemia
IDDM: Insulin-dependent diabetes mellitus
ICU: Intensive care unit
KD: Kidney disease
LDH: Lactate dehydrogenase
MI: Myocardial infarction
MPG: Mean pressure gradient
NYHA: New York heart Association
PAD: Peripheral artery disease
POD: Postoperative delirium
PPI: Permanent pacemaker implantation
PM: Pacemaker
PPG: Peak pressure gradient
PVL: Paravalvular leakage
RDAVR: Rapid deployment aortic valve replacement
RF: Radial forces
SB: Sutureless bioprosthesis
SD: Standard deviation
SFS: Standard full sternotomy
SVD: Structural valve deterioration
SUAVR: Sutureless aortic valve replacement
SS: Stainless steel
TAVI: Transcatheter aortic valve implantation
TAVR: Transcatheter aortic valve replacement
TEE: Transesophageal echocardiography
TTE: Transthoracic echocardiography
